# Interleukin pathway genotype and the grandiosity dimension of psychosis: a machine learning gene set analysis with unsupervised anomaly detection controls

**DOI:** 10.3389/fimmu.2026.1936750

**Published:** 2026-10-16

**Authors:** Maksymilian A. Brzezicki, Przemysław T. Zakowicz, Paweł Kapelski, Monika Wilkość-Dębczyńska, Aleksandra Rajewska-Rager, Maria Skibińska

**Affiliations:** 1 Department of Neural Engineering and Space Medicine, Institute of Medical Sciences, University of Zielona Góra, Zielona Góra, Poland; 2 Jesus College, University of Oxford, Oxford, United Kingdom; 3 Space Research Centre, Polish Academy of Sciences, Warsaw, Poland; 4 Department of Genetics in Psychiatry, Poznań University of Medical Sciences, Poznań, Poland; 5 Department of Psychology, Kazimierz Wielki University, Bydgoszcz, Poland

**Keywords:** anomaly detection, cytokine genetics, gene-set analysis, grandiose delusions, grandiosity, interleukin-10, psychosis, symptom dimensions

## Abstract

Immune involvement in psychosis is well supported, although most of the evidence comes from studies of negative and deficit symptoms. Grandiosity, in comparison, has received little attention. It is, however, the one symptom for which the two main accounts of inflammation in psychosis, activation and reward blunting, make opposite predictions. We tested these predictions in 489 patients with schizophrenia from the Poznań cohort, genotyped for 26 functional variants in eight interleukin-pathway genes and rated with the Operational Criteria Checklist (OPCRIT). Grandiosity was scored from the two OPCRIT items for inflated self-esteem and grandiose delusions. The panel was first tested as a whole, and each gene was then analyzed as a set with the sequence kernel association test (SKAT), with false-discovery-rate control and selection-adjusted permutation. For any gene that passed, we localized the signal within it and estimated the effect with Firth penalized logistic regression. Its stability was examined by held-out splits, jackknife, fragility analysis, comparison with five other symptom dimensions, and anomaly detection. The panel as a whole did not predict grandiosity (out-of-fold Spearman rho = 0.041, permutation p = 0.159). One gene, IL10, showed an association (SKAT p = 0.0028, q = 0.023; selection-adjusted p = 0.025), which was confined to the −819/−592 promoter locus. Carriers of two copies had a Firth odds ratio of 5.28 (95% CI 2.37 to 12.48, p = 4.5 × 10^-5^), consistent with a recessive effect, and 65% of the 26 carriers had grandiosity, against 24% of non-carriers. The effect was specific to grandiosity (standardized beta 0.19, p = 2 × 10^-5^) and moderately reproducible across split halves (r = 0.36; mania control r = 0.03). It did not depend on sex, age, or age at onset, and it was unchanged under alternative SKAT kernels, under panel principal components, and after adjustment for neuroleptic response. Two-copy carriage did not differ between the 466 cases and 482 controls. We propose a recessive IL10 −819/−592 effect specific to grandiosity as a hypothesis for independent replication.

## 1 Introduction

Immune dysregulation is a consistent finding in psychosis. The first evidence came from epidemiology. Children born to mothers who had an infection during pregnancy have an increased risk of psychotic illness in later life (1, 2), which suggests that the immune system influences brain development before birth. The genetic evidence points the same way. The strongest common signal for schizophrenia lies in the major histocompatibility complex and is largely explained by variation in complement component 4; risk alleles increase its expression, and complement component 4 marks synapses for elimination by microglia (3). Most other risk loci involve neuronal and synaptic genes (4); the immune contribution to risk is therefore partial. In adults with psychosis, circulating cytokines are reproducibly altered. Some rise during acute episodes and normalize with treatment, whereas others remain elevated regardless of clinical state, a pattern of state and trait markers (5, 6) that has led to the proposal of an immunophenotype of psychosis (7). Whether these immune changes cause psychosis or accompany it is not known. If inflammation drove symptoms, reducing it should relieve them. In randomized trials, however, anti-inflammatory drugs added to antipsychotic treatment have had modest and inconsistent effects (8).

The immune signal has also been linked to the clinical picture, mainly to negative, deficit, and paranoid symptoms (9, 10). The cytokines involved form a small network of pro-inflammatory and anti-inflammatory mediators. Among the pro-inflammatory cytokines, interleukin-1α and interleukin-1β rise in acute illness and normalize with treatment (5), and interleukin-1β is raised in the cerebrospinal fluid of first-episode patients (11). Both are opposed by the interleukin-1 receptor antagonist, whose gene has been studied in this cohort (12). Interleukin-6 is among the cytokines most consistently elevated in acute psychosis (5). Mendelian randomization implicates interleukin-6 signalling in schizophrenia, with higher C-reactive protein associated with lower risk (13), and variants in the receptor gene have been associated with the disorder in this cohort (14). Interleukin-10 is the principal anti-inflammatory cytokine of the network. In meta-analysis, its promoter polymorphisms carry a small increase in schizophrenia risk (15). Transforming growth factor β1 regulates the balance between effector and regulatory T cells and is also raised in acute psychosis and normalizes with treatment (5).

Grandiosity has been largely absent from this work, even though it is remarkably common. Grandiose beliefs, defined as an inflated sense of one’s power, knowledge, identity, or worth, occur in approximately half of people with psychosis and in two thirds of those with mania (16). Even so, they have been studied far less than hallucinations or persecutory delusions, and their biology is essentially unknown. The few immune studies to date have measured circulating cytokines (17). Grandiosity is nonetheless informative, because the two main hypotheses linking inflammation to behavior predict opposite associations. Grandiosity is a defining feature of mania, and acute mania is among the more clearly pro-inflammatory states in psychiatry (6). A pro-inflammatory constitution should therefore increase the prevalence of grandiosity if inflammation drives activation. The opposite prediction follows if inflammation suppresses striatal dopamine signalling and causes anhedonia and psychomotor slowing (18). Negative symptoms are predicted by both hypotheses and do not discriminate between them, whereas grandiosity does.

The two hypotheses need not be exclusive, as inflammatory pathways may act through a net balance between pro-inflammatory and anti-inflammatory cytokines. To test this possibility, we assembled a panel of genes covering both classes of cytokine and their receptors and antagonists. Each gene had been implicated in psychosis independently. The aim was to index a person’s constitution. The panel was therefore restricted to functional promoter, coding, and regulatory variants, so that each locus reflects a stable difference in cytokine production. Grandiosity is the clearest behavioral expression of activation and the symptom on which such a set-point should be most visible. We tested the panel as a whole against grandiosity first and examined its components only afterwards.

## 2 Materials and methods

### 2.1 Participants, recruitment, and ethics

Participants were drawn from the Poznań psychiatric-genetics cohort, in which patients with a clinical diagnosis of schizophrenia were recruited and phenotyped as described previously (12, 14, 19, 20). Diagnosis and symptom rating used the Operational Criteria Checklist for Psychotic Illness (OPCRIT; 21). This analysis includes the subset of the cohort with both interleukin genotyping and item-level OPCRIT data.

The interleukin panel and the genotyping come from the same group’s case-control study of this Polish cohort (14). After exclusion of records with incomplete genotype or phenotype data, 502 patients with schizophrenia and 521 healthy controls were available. All were recruited at a single site and were of Polish origin, as in the source study. The present analysis uses the schizophrenia cases with item-level OPCRIT data. No genome-wide genotyping exists for this cohort, so ancestry principal components could not be computed; the stratification checks that the panel itself permits are described in Section 2.4. Patients were recruited through the Psychiatric Genetics Unit, Department of Psychiatry, Poznań University of Medical Sciences, and diagnosed according to Diagnostic and Statistical Manual of Mental Disorders, Fourth Edition (DSM-IV) criteria validated with OPCRIT. All participants gave written informed consent. The study was approved by the Local Bioethics Committee of Poznań University of Medical Sciences (approval no. 149/10).

Of the linked sample, 490 patients had both genotype and OPCRIT data. Of these, 489 had both grandiosity items observable and formed the analysis sample; one patient was excluded for a missing grandiosity item. Characteristics of the analysis sample are given in **Table 1**. Controls in the genotype file have genotypes only, with no symptom data. They were used for Hardy–Weinberg checks and as an allele-frequency reference. They also served the case-control comparison of the −819/−592 locus (Section 3.5).

**Table 1:** Characteristics of the sample.

Characteristic	Value
N linked, genotyped (schizophrenia)	490
N analysis sample (grandiosity observable)	489
Male, n (%)	223 (45.6)
Female, n (%)	266 (54.4)
Age at assessment, years, mean (SD)	31.6 (11.4)
Age at onset, years, mean (SD)	23.1 (6.4)
Grandiosity (0 to 4 sum), mean (SD)	0.73 (1.33)
Any grandiosity (score >0), n (%)	133 (27.2)
Manic-cluster dimension, mean (SD)	0.21 (0.30)
Depressive dimension, mean (SD)	1.18 (0.86)
Negative dimension, mean (SD)	1.00 (0.64)
Other-delusions dimension, mean (SD)	0.52 (0.25)
Hallucinations dimension, mean (SD)	0.40 (0.28)

Grandiosity is the sum of OPCRIT items 56 and 57 (range 0 to 4); the manic-cluster, depressive, negative, other-delusions, and hallucinations dimensions are the mean of their constituent OPCRIT item ratings, grouped following the dimensional approach validated in this cohort (19). Higher values indicate greater severity.

### 2.2 Outcome and covariates

The primary outcome was grandiosity, defined as the sum of two OPCRIT items, inflated self-esteem (item 56) and grandiose delusions (item 57). Each item is rated 0 to 2, giving a score of 0 to 4. Missing OPCRIT items were left missing, and a patient missing either grandiosity item was excluded from outcome analyses; the outcome was never imputed. Two forms of the outcome were used, and the form is stated with each analysis. For the aggregate prediction models and the gene-set screen, the score was transformed to normality with a rank inverse-normal (Blom) transform. For the localized effect-shape and effect-size estimates, the outcome was the presence of any grandiosity (score above zero). The score is zero in 73% of patients and the effect proved recessive. The recessive contrast is also reported on the continuous score (Section 3.3).

OPCRIT ratings are lifetime ratings. Item 2 (period rated) was coded lifetime ever for 441 of the 489 patients (90.2%), current episode for 44, and worst episode for 3. The grandiosity items therefore record whether the symptom was ever present. They do not measure its intensity at interview. Treatment status at assessment and drug-naive status were not recorded. Item 89 (resolution of psychotic symptoms on neuroleptics; 394 yes, 91 no, four missing) was used in a sensitivity analysis (Section 2.4). Covariates were sex (OPCRIT item 3, coded 0 for male and 1 for female in the cohort data dictionary), age at assessment, and age at onset (OPCRIT item 4). Missing covariate values were replaced with the covariate median, and covariates were standardized before entry. For the specificity analysis, five further symptom dimensions were computed as the mean of their constituent OPCRIT items: a manic cluster, a depressive dimension, a negative dimension, an other-delusions dimension, and a hallucinations dimension. Six dimensions were therefore examined: grandiosity as the primary outcome and five comparison dimensions. Items 56 and 57 do not enter any of the five comparison dimensions, including the manic cluster. The comparison dimensions were used to test whether any interleukin effect was specific to grandiosity.

### 2.3 Genotyping, the interleukin panel, and quality control

The panel comprised 26 markers in eight interleukin-pathway genes: IL1A (rs1800587, rs17561), IL1B (rs1143643, rs16944, rs1143623, rs1143633, rs1143627), IL1RN (an 86 bp variable-number-tandem-repeat plus rs419598, rs315952, rs9005, rs4251961), IL6 (rs1800797, rs1800795), IL6R (rs2228145, rs4537545, rs4845617), IL10 (rs1800871, rs1800872, rs1800896, rs1800890, rs6676671), IL10RA (rs2229113, rs3135932), and TGFB1 (rs1800469, rs1800470). Genotyping used TaqMan single-nucleotide polymorphism (SNP) genotyping assays (Applied Biosystems, Foster City, CA, USA), as in the same group’s earlier genotyping of this cohort (14). Gene symbols (IL10) refer to the gene and its markers; spelled-out names (interleukin-10) refer to the protein. Marker-level call rates, minor-allele frequencies in cases and controls, Hardy–Weinberg tests, and allelic case-control tests for all 25 SNPs are given in **Supplementary Table S1**, and pairwise linkage disequilibrium (LD; r²) within each gene set in **Supplementary Table S2**.

Each SNP was coded as the number of minor alleles carried (0, 1, or 2), with the minor allele defined in this sample. The IL1RN repeat was encoded as its short-repeat count. Missing dosages, 169 of 12,714 genotype cells (1.33%), were mean-imputed once within the analysis sample before any model was fitted. The imputation uses no outcome information, so it cannot carry outcome information between cross-validation folds (Section 2.4). The IL10 −819/−592 locus was formed from the two promoter tag SNPs rs1800871 (−819) and rs1800872 (−592). These are in near-complete linkage disequilibrium in this sample (dosage correlation r = 0.994) and were therefore treated as a single locus. Their summed minor-allele dosage was collapsed into 0, 1, or 2 copies of the shared −819/−592 haplotype. The direction of the effect is taken empirically from the sample and labelled by promoter position. It is not asserted to be the functional low-producer haplotype, because the canonical −1082 variant (rs1800896) is null in these data (Section 3.3). Quality control for the two IL10 tag SNPs comprised the genotyping call rate and a Hardy–Weinberg equilibrium test (chi-square goodness of fit against expected genotype frequencies), in cases and separately in the independent controls in the genotype file; the results are reported in Section 3.1. The genetic association analyses follow the STrengthening the REporting of Genetic Association studies (STREGA) guideline (22).

### 2.4 Statistical analysis

The analysis was designed for a panel in which no single marker is expected to carry a detectable effect. Evidence was aggregated within genes. Each gene was tested as a variance component, which allows variants with opposite effects to contribute to the same test. A gene was accepted only if it passed a selection-adjusted threshold, reproduced across random split halves, and remained associated in patients who had not been used to select it. This approach of combining many weak signals into an aggregate index that must then reproduce on independent data is the same principle used in condition monitoring in engineering (23, 24). The panel was also examined without reference to grandiosity, by identifying patients whose overall genotype profile was unusual and asking whether they differed in grandiosity (Section 2.5); this corresponds to anomaly detection in the same field (25). The whole analysis is gated: the pre-specified aggregate and latent tests are performed first, and the single-locus analysis is reported as conditional on their result.

Ridge regression was used to predict grandiosity from the panel and covariates, and from covariates alone, in five-fold cross-validation with the penalty chosen within each training fold. The two models were compared on their out-of-fold predictions. Performance was the Spearman correlation between out-of-fold predictions and the outcome, tested against a permutation null. Genotype structure was summarized by principal component analysis. As a further check, several supervised classifiers for the presence of any grandiosity (logistic regression, elastic-net logistic regression, random forest, gradient boosting, and a support vector machine) were evaluated by pooled out-of-fold receiver-operating-characteristic curves under ten-fold stratified cross-validation. The prediction models are reported in line with the Transparent Reporting of a multivariable prediction model for Individual Prognosis Or Diagnosis, updated for regression and machine learning methods (+AI) (TRIPOD+AI) guideline (26).

Each gene was tested as a set against the rank inverse-normal grandiosity score, adjusted for covariates, with the sequence kernel association test (SKAT). SKAT is a variance-component score test that aggregates a gene’s variants into a single statistic and retains power when variants act in opposing directions (27). The kernel was linear with equal variant weights, so that each gene’s statistic is the sum of squared score contributions of its minor-allele dosages. Equal weights are the appropriate default for a panel of common functional variants. The Beta(1,25) minor-allele-frequency weights proposed for rare-variant sequencing data would have concentrated the test on the rarest markers. That is inappropriate here, because the two-copy carriers at the −819/−592 locus are homozygous for a common allele (minor-allele frequency 0.23). The test accommodates a variable number of markers per gene and correlation between markers through its null distribution, an eigenvalue-weighted mixture of chi-square variables computed from each gene’s own marker matrix, so correlated markers are not counted as independent. As sensitivity analyses, the screen was repeated with Beta(1,25) weights, with standardized dosages, and on an LD-pruned panel from which the second member of every within-gene pair with r² above 0.8 was dropped (**Supplementary Table S3**). The observed per-gene p value used the exact distribution of the quadratic form (28); inside permutation loops the four-moment chi-square approximation was used for speed (29). Multiplicity across the eight genes was controlled by the Benjamini–Hochberg false-discovery rate (30). The gene reported is the one with the smallest of eight p values, so its significance was also assessed by selection-adjusted permutation: grandiosity was shuffled, the whole eight-gene screen was re-run on each shuffle, and the observed minimum p value was compared with the minimum obtained in each shuffle. The naive per-gene permutation is reported alongside for comparison. The distribution of the winning gene across permutations was inspected to check that the screen did not favor any gene.

To determine the shape of the effect, additive, recessive, and saturated logistic models for the presence of any grandiosity were compared by the Akaike information criterion. The recessive contrast compared two-copy carriers with all other patients. Because the carrier group was small, the odds ratio was estimated with Firth penalized logistic regression, which reduces small-sample bias and remains stable when the outcome nearly separates the groups (31, 32). A profile-likelihood confidence interval and a penalized likelihood-ratio test were computed, both unadjusted and adjusted for covariates. The fragility of the recessive estimate was quantified in two ways: as the number of grandiose two-copy carriers that would need to be re-coded as non-grandiose, and separately removed from the sample, before the penalized likelihood-ratio p exceeded 0.05; and as the range of the odds ratio over 300 random leave-k-out subsets of carriers for k = 1 to 5. The recessive contrast was also estimated on the continuous rank inverse-normal score by ordinary least squares and on the raw score by the Mann–Whitney test.

Selection was separated from evaluation with a repeated discovery and held-out split. On each split, the best gene was chosen on one random half, and its gene-set p value and the direction of the two-copy effect were evaluated on the other half. A jackknife over the two-copy carriers checked that no single carrier drove the effect. Confounding was checked by comparing two-copy carriers with non-carriers on sex, age, and age at onset (Mann–Whitney). Specificity was assessed by regressing each of the six symptom dimensions on IL10 −819/−592 dose, adjusted for covariates, and comparing the standardized coefficients. Split-half reproducibility was quantified as the correlation of per-marker effect estimates between random halves of the sample, computed for grandiosity and, as a control, for the manic cluster, each against a permutation null. Standardization, the ridge penalty, and the per-marker effect estimates for the cross-fitted display were fitted inside training folds only, so that no held-out patient influenced its own prediction. The one exception is the unsupervised mean imputation of missing dosages described in Section 2.3, which was applied once across the analysis sample. In the absence of genome-wide data, two checks that the panel allows were run instead. The first five principal components of the 26-marker genotype matrix were compared between cases and controls, since a difference would indicate that the two groups were not drawn from the same population. The IL10 gene-set test and the recessive odds ratio were then re-estimated with the first five components of the 24 non-IL10 markers added as covariates. The recessive estimate was also re-run with OPCRIT item 89 added as a covariate and within each of its strata.

Model settings were fixed in advance and not tuned to the outcome. Ridge penalties were selected by cross-validation over a logarithmic grid from 10^-^² to 10³. The elastic-net classifier used an L1 ratio of 0.5, inverse-regularization C of 0.5, and the SAGA solver; the random forest used 300 trees; gradient boosting and the support vector machine used library defaults, with a radial-basis kernel for the latter. Five-fold cross-validation was used for the out-of-fold prediction and ten-fold cross-validation for all other analyses; folds were stratified by outcome when the outcome was binary. Analyses were run in Python 3.12.3 with numpy 2.4.4, pandas 3.0.2, scipy 1.17.1, scikit-learn 1.8.0, and statsmodels 0.14.6. The main analysis, the sensitivity and supplementary analyses, and the anomaly-detection analysis are in Jupyter notebooks that reproduce every number and figure in the manuscript from fixed seeds (Data availability statement).

### 2.5 Unsupervised anomaly detection

The data were also examined from the unsupervised direction. Four detectors were applied: an isolation forest (33); a one-class support vector machine with a radial-basis kernel (34); a fully connected neural autoencoder of size 26-16-4-16-26 (three hidden layers with a four-unit bottleneck, rectified-linear activations, and a linear output), trained with the Adam optimizer to reconstruct its input under an L2 weight penalty (alpha = 0.001) for up to 3,000 iterations from a fixed seed and scored by mean squared reconstruction error (35); and, as a linear reference, principal-component reconstruction error with eight components. The isolation forest used 400 trees; the one-class support vector machine used nu = 0.05 with gamma set by the scale heuristic. Inputs were the 26 dosages, median-imputed and standardized. Each score was scaled so that higher values indicated a more unusual genotype profile, and the four scores were combined by rank averaging into a single ensemble score. Patients were classified as unusual on this score without reference to grandiosity. We then tested whether unusual patients differed in grandiosity from the rest, and whether any difference was attributable to the IL10 locus. A four-unit bottleneck on 26 inputs cannot represent the identity map, so the autoencoder cannot reduce to an identity mapping. Its reconstruction error was nonetheless compared, in-sample and under five-fold cross-validation, with the principal-component reference at the same dimensionality; the bottleneck (2, 4, or 8 units) and penalty (0.001 or 0.01) were varied; and the ensemble was recomputed without the autoencoder (**Supplementary Table S6**).

## 3 Results

### 3.1 Sample characteristics and genotyping quality

The sample was 45.6% male and 54.4% female, with a mean age of 31.6 years and a mean age at onset of 23.1 years. Mean scores on the depressive, negative, delusional, and hallucinatory dimensions were modest (**Table 1**). Grandiosity was present (score above zero) in 27.2% of patients, and 10.4% scored the maximum of 4. The distribution was bimodal (**Supplementary Table S4**): 356 patients (72.8%) scored 0, 18 (3.7%) scored 1, 56 (11.5%) scored 2, 8 (1.6%) scored 3, and 51 (10.4%) scored 4. Grandiose delusions (item 57) were rated in 110 patients (22.5%) and inflated self-esteem (item 56) in 100 (20.4%). Call rates for the 25 SNPs were 93.5% to 100%. No marker departed from Hardy–Weinberg equilibrium in cases or in controls, and no marker differed in allele frequency between cases and controls after false-discovery-rate correction (**Supplementary Table S1**). Eight within-gene marker pairs had r² above 0.8, including the two IL10 promoter tags (r² = 0.988); the TGFB1 pair rs1800469 and rs1800470 had r² = 0.764 (**Supplementary Table S2**).

### 3.2 Panel-level tests and gene-set screen

The pre-specified aggregate and latent tests were null. Out-of-fold prediction from the full panel plus covariates was no better than from covariates alone (Spearman rho = 0.041 versus 0.028; permutation p = 0.159). Genotype structure showed no dominant axis; the first two principal components explained 17.5% and 14.0% of variance. None of the supervised classifiers for any grandiosity performed better than chance (areas under the curve 0.51 to 0.55; **Figure 1**, top row). At panel level, therefore, interleukin genotype did not predict grandiosity. The analyses that follow ask whether a signal exists within the panel.

**Figure 1:**
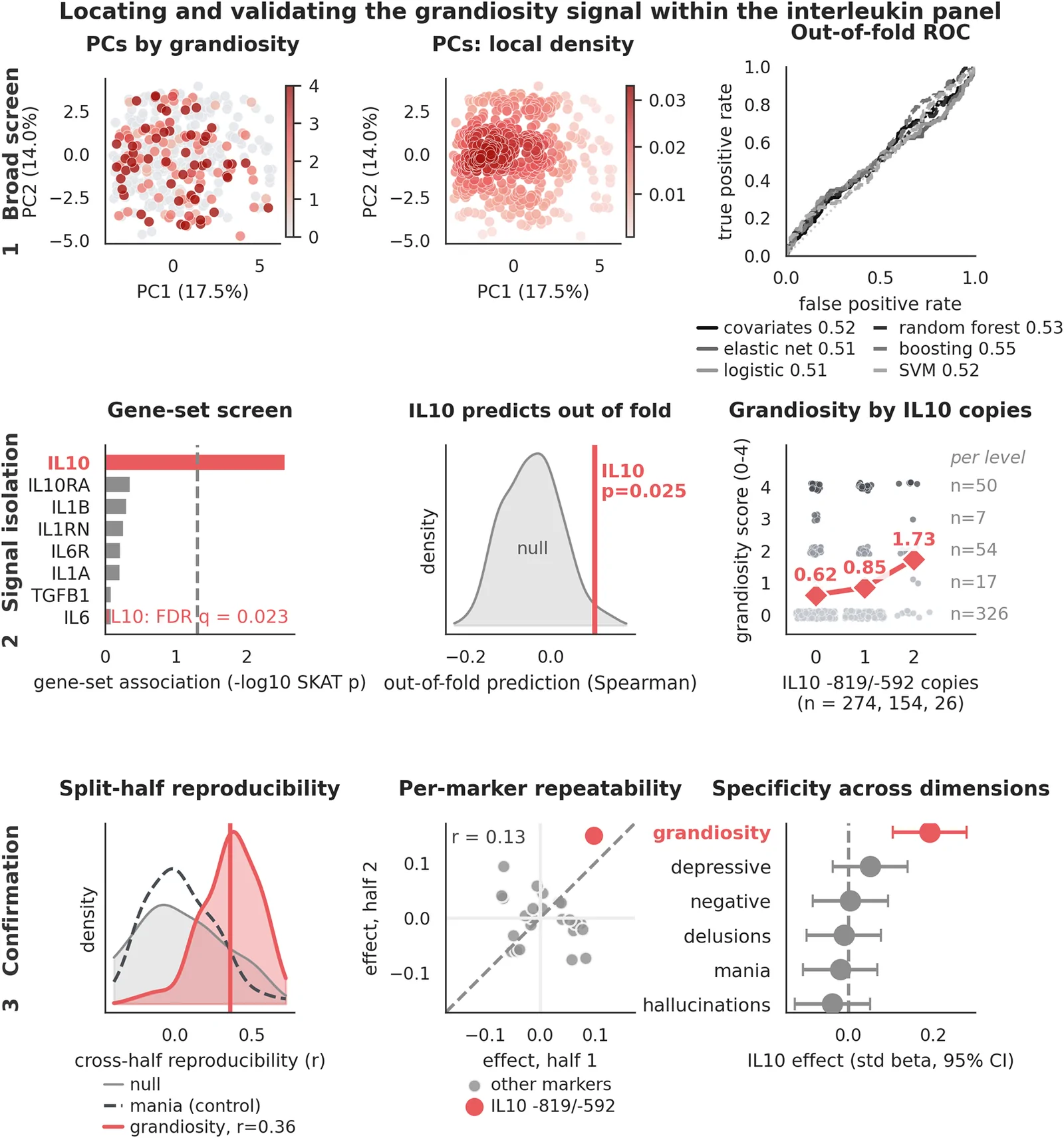
Location and validation of the grandiosity signal within the interleukin panel. Top row, panel-level tests: the first two genotype principal components, colored by grandiosity, show grandiose patients scattered through a single cloud with no genetic subgroup and no grandiosity axis, and no supervised classifier separates patients with and without grandiosity (all out-of-fold areas under the curve near 0.5). Middle row, gene-set screen: when variants are aggregated within genes, IL10 is the only gene that crosses the false-discovery threshold; the IL10 out-of-fold predictor lies beyond its permutation null; and grandiosity rises in two-copy carriers of the −819/−592 locus but not in one-copy carriers (group means 0.62, 0.85, 1.73; per-level counts shown). Bottom row, robustness: per-marker effect estimates computed in two random halves of the sample correlate for grandiosity (mean r = 0.36) but not for the manic-cluster control (mean r = 0.03); the two −819/−592 tags are the outlying markers in the per-marker comparison between halves; and the IL10 −819/−592 dose effect is present for grandiosity and near zero for the five other symptom dimensions.

Tested gene by gene as sets, seven of the eight genes were non-significant (SKAT p = 0.44 to 0.81; all false-discovery-rate q = 0.81; **Figure 1**). IL10 was the exception (SKAT p = 0.0028) and the only gene to survive false-discovery-rate control (q = 0.023; **Figure 1**, middle row). The selection-adjusted permutation, which re-ran the whole eight-gene screen on each shuffle, gave p = 0.025; the naive per-gene permutation gave p = 0.0040. Under the null, the permutation screen selected each gene in approximately one eighth of shuffles, so the screen did not favor any gene. The result did not depend on the kernel or weighting (**Supplementary Table S3**). With Beta(1,25) minor-allele-frequency weights the IL10 gene-set p was 0.0003 (q = 0.002), with standardized dosages 0.0012 (q = 0.010), and on the LD-pruned panel 0.0052 (q = 0.042). Under every variant, IL10 was the only gene to survive false-discovery-rate control.

### 3.3 Localization, shape, and size of the effect

Within IL10, the association was confined to the two promoter tags. Both were positively associated with grandiosity (rs1800871 at −819, r = 0.17; rs1800872 at −592, r = 0.18; both p = 0.0001). Across all 26 panel markers, these were the only two to survive false-discovery-rate correction (q = 0.001 each). The −1082 variant was null and opposite in sign (rs1800896, r = −0.08, p = 0.09), as were the two remaining IL10 markers (both p >0.12). Leaving markers out confirmed the localization: the IL10 gene-set p rose from 0.0028 to 0.082 when the −819/−592 tags were removed and fell to 0.0002 when only those two tags were retained. The effect is therefore a property of the −819/−592 locus itself.

Mean grandiosity score was 0.62 with no copies, 0.85 with one copy, and 1.73 with two, and the proportion with any grandiosity was 24%, 30%, and 65% (**Figure 1**). Model comparison favored a recessive shape (Akaike information criterion 527.5 for recessive, 527.6 for saturated, 530.1 for additive). The one-copy contrast was indistinguishable from null (odds ratio 1.37, 95% CI 0.88 to 2.13, p = 0.16), and the two-copy contrast was large (odds ratio 6.07, 95% CI 2.58 to 14.27, p <0.0001). The Firth-penalized recessive estimate, used because of the small carrier cell, gave an odds ratio of 5.28 adjusted for covariates (95% profile confidence interval 2.37 to 12.48; penalized likelihood-ratio p = 4.5 × 10^-5^) and 5.25 unadjusted (2.36 to 12.37). The interval is wide because there are only 26 carriers. The same contrast on the continuous score gave a difference of 0.58 SD in the rank inverse-normal score (p = 5 × 10^-5^) and a Mann–Whitney p of 2 × 10^-5^ on the raw score (Cliff’s delta 0.39), so the effect does not depend on dichotomization. Of the 26 carriers, 17 were grandiose. Six of them would have to be re-coded as non-grandiose (fragility index 6; odds ratio at that point 2.11, p = 0.073), or nine removed from the sample, before the penalized likelihood-ratio test lost nominal significance. Over 300 random subsets removing up to five carriers, the odds ratio ranged from 3.62 to 10.73 and remained significant in every subset. The full genotype-by-score counts are given in **Supplementary Table S5**.

### 3.4 Specificity, reproducibility, and confounding

Regressed on IL10 −819/−592 dose, grandiosity showed a standardized effect of 0.19 (95% CI 0.10 to 0.28, p = 2 × 10^-5^). The manic-cluster, depressive, negative, delusional, and hallucinatory dimensions were all near zero (standardized beta −0.02, 0.05, 0.00, −0.01, and −0.04; every interval includes zero and all p >0.25; **Figure 1**). A locus acting as a marker of overall severity would be expected to spread across dimensions; this one does not. Per-marker effects correlated moderately across random split halves for grandiosity (mean r = 0.36, positive in 96% of splits) but not for the mania control (mean r = 0.03, positive in 51%). This shows that the sign is consistent across halves; it does not show strong reproducibility. In the repeated discovery and held-out split, IL10 was selected on the discovery half in 83% of splits, against a chance rate of 12.5%; the two-copy effect kept the same direction on the held-out half in 100% of 500 evaluable splits; and the selected gene reached nominal significance on the held-out half in 43% of splits (median held-out p = 0.070). Removing any one of the 26 carriers in turn left the odds ratio between 4.93 and 5.96, so no single carrier accounts for the effect. Carriers and non-carriers did not differ in sex, age, or age at onset (Mann–Whitney p = 0.47, 0.69, and 0.93). Panel principal components did not separate cases from controls (first five components, Mann–Whitney p = 0.13 to 0.46). Adding the first five components of the 24 non-IL10 markers as covariates left the IL10 gene-set test (p = 0.0032) and the recessive odds ratio (5.32, 95% CI 2.38 to 12.63, p = 4.2 × 10^-5^) essentially unchanged. Adjustment for OPCRIT item 89 gave an odds ratio of 5.78 (95% CI 2.55 to 13.95, p = 2.4 × 10^-5^, n = 450). The excess of grandiosity among carriers was present both in patients whose psychotic symptoms resolved on neuroleptics (65% versus 29%, 20 carriers) and in those whose symptoms did not (67% versus 11%, 6 carriers). The same pattern is visible at the level of individual patients in the cross-fitted contribution map (**Figure 2**): the two −819/−592 tags contribute strongly in patients with high grandiosity, and the other 24 markers show no structure.

**Figure 2:**
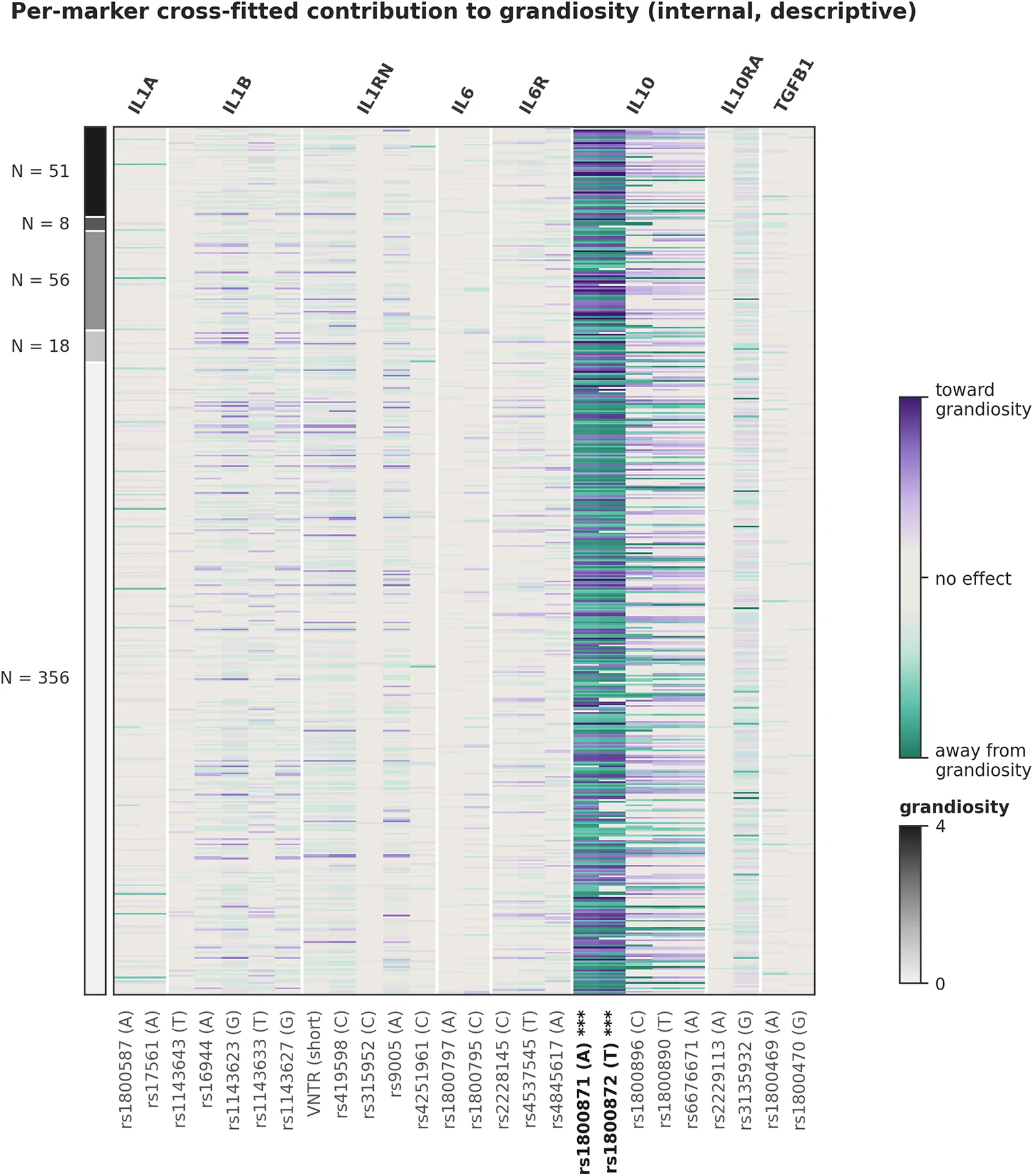
Cross-fitted per-patient contribution across the interleukin panel. Each row is one patient (n = 489), ordered by grandiosity score from high (top) to low, shown in the left strip (darker is higher; counts per level indicated). Each column is one of the 26 markers, grouped by gene (labels above). Cell color is the cross-fitted contribution of that marker to the patient’s predicted grandiosity, with the per-marker effect learned only on training folds so that no patient contributes to its own row. Only the two IL10 promoter tags, rs1800871 (−819) and rs1800872 (−592), survive false-discovery-rate correction across the 26 markers (marked); their columns carry a concentrated contribution in high-grandiosity patients, and the remaining panel shows no structure.

### 3.5 Unsupervised anomaly detection and case-control comparison

The four detectors agreed well enough to be combined. The isolation forest and one-class support vector machine correlated at Spearman 0.88, and each correlated with the two reconstruction-based methods at 0.33 to 0.52 (**Figure 3**). The ensemble anomaly score was unrelated to grandiosity (Spearman −0.034, p = 0.45), and the most anomalous decile of patients had slightly less grandiosity than the rest (any grandiosity 20% versus 28%). The IL10 −819/−592 two-copy carriers were flagged as more anomalous, as expected for a rare genotype (mean anomaly percentile 72 versus 48 for non-carriers; Mann–Whitney p < 0.0001), but their grandiosity was mixed, so anomaly status did not account for the grandiosity signal. The anomalies were instead driven by rare genotypes at IL6R, IL1RN, IL10RA, and TGFB1, and removing the five IL10 markers left the null anomaly-to-grandiosity association unchanged for every detector. The autoencoder behaved as intended. It converged in 434 iterations. Its reconstruction error was close to that of the principal-component reference at the same dimensionality (in-sample mean squared error 0.478 against 0.502 for four components), and under five-fold cross-validation the out-of-sample error was only slightly higher (0.536); the network therefore neither overfitted nor collapsed to an identity map. Its anomaly score remained uncorrelated with grandiosity when the bottleneck was set to 2, 4, or 8 units and the penalty to 0.001 or 0.01 (Spearman rho −0.06 to −0.005), and an ensemble built without the autoencoder gave the same null result (rho = −0.023, p = 0.62; **Supplementary Table S6**).

**Figure 3:**
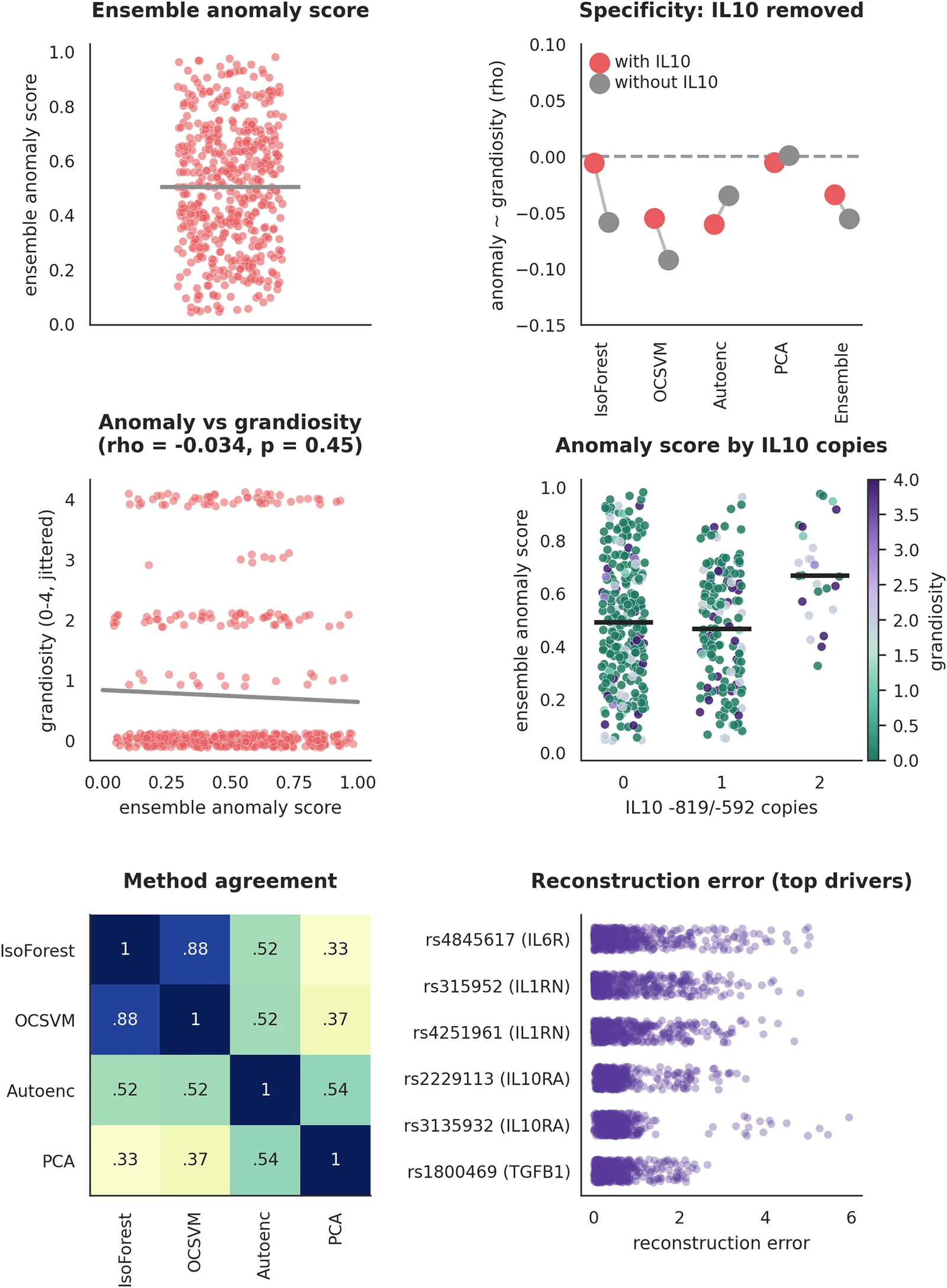
Unsupervised anomaly detection and grandiosity. Top row: the ensemble anomaly score for every patient (left); and the anomaly-to-grandiosity correlation for each detector with the IL10 markers included (red) and removed (gray), near zero throughout (right). Middle row: anomaly score against grandiosity (left, one point per patient, grandiosity jittered); and anomaly score by IL10 −819/−592 copy number, each point colored by grandiosity on a teal-to-purple scale (right). Bottom row: agreement between the four detectors as a correlation matrix (left); and per-marker reconstruction error for the markers that most drive the anomalies, at IL6R, IL1RN, IL10RA, and TGFB1 rather than at the IL10 −819/−592 locus (right); the error shown is the mean of the autoencoder and principal-component reconstruction errors. IsoForest, isolation forest; OCSVM, one-class support vector machine; Autoenc, autoencoder; PCA, principal-component reconstruction.

A locus that marks a symptom dimension within the illness should not distinguish cases from controls. In the 466 genotyped cases and 482 controls with the locus called, copy-number distributions were similar (two-copy carriers 5.6% versus 5.2%; genotypic chi-square p = 0.15; additive odds ratio per copy 0.86, 95% CI 0.70 to 1.07, p = 0.17; Firth recessive odds ratio for case status 1.08, 95% CI 0.62 to 1.90, p = 0.79), and no panel marker differed between cases and controls after false-discovery-rate correction (**Supplementary Table S1**). Two-copy carriage was, however, more frequent among the 128 grandiose cases with the locus called than among controls (13.3% versus 5.2%; Firth recessive odds ratio 2.82, 95% CI 1.46 to 5.32, p = 0.0024). In this cohort, therefore, the locus behaves as a marker of a symptom dimension within schizophrenia. It does not behave as a susceptibility marker for the diagnosis. This is in keeping with the modest susceptibility effects reported for IL10 promoter variants in meta-analysis (15).

## 4 Discussion

Constitutional interleukin genotype did not predict grandiosity at panel level, but one locus within the panel did. Two-copy carriers of the IL10 −819/−592 promoter pair, approximately 5% of patients, had grandiosity about three times as often as non-carriers. The effect was recessive and confined to grandiosity; none of the other five symptom dimensions showed it. Its direction was the same in random split halves and in held-out subsamples. Sex, age, and age at onset did not explain it. It survived alternative SKAT kernels and adjustment for panel principal components, and it was unchanged after adjustment for neuroleptic response. The locus did not differ between cases and controls. The effect is nonetheless small and rests on 26 carriers. We therefore present it as a hypothesis for replication.

Parts of this result are consistent with previous work. IL10 promoter variation carries a small susceptibility signal for schizophrenia in meta-analysis (15). The −592 variant that anchors our locus has been related to cognition in first-episode patients (36). A clinically relevant effect at this promoter is therefore not unexpected. The dimension on which it appears is also explicable. IL10 variation has been examined alongside dopamine-pathway genes: independently of the dopamine D4 receptor in relation to vulnerability (37), in combination with dopamine beta-hydroxylase in relation to general symptoms (38), and with catechol-O-methyltransferase in relation to cognition (39). Of all psychotic symptoms, grandiosity is the one most closely tied to dopaminergic reward activation. An association between an anti-inflammatory cytokine gene and this dimension is therefore consistent with that literature. The size of the effect is in keeping with the modest germline-to-phenotype effects reported elsewhere (40).

Other findings are harder to reconcile. The same −592 variant has been tied to negative and deficit symptoms of schizophrenia, with the low-expression alleles carrying greater burden (41), whereas we implicate the locus in grandiosity, a symptom of activation. A Saudi study found no association of IL10 promoter genotype with symptoms (42). At the level of circulating protein the picture differs again. In first-episode psychosis, interleukin-10 tracked overall severity and disturbance of volition; it did not track grandiosity. Pro-inflammatory interleukin-1β correlated inversely with the grandiosity item in the same sample of 38 patients (17). The −1082 low-producer variant, emphasized in some susceptibility work, was null here.

Several factors could account for these discrepancies. The studies differ in ancestry and in the instruments, symptom dimensions, and methods used. A pleiotropic locus may also be detected on different symptom dimensions in different samples. State and germline measures also index different quantities, phasic protein and tonic set-point. Reverse causation affects the protein studies but not this one, so a protein signal for severity and a germline signal for grandiosity are not contradictory. Direction is a further source of apparent conflict. Because we label the locus by position and do not assert the functional low-producer direction, disagreements with functional-allele studies may reflect haplotype and linkage-disequilibrium differences and need not indicate true opposition. The design aggregated evidence within genes and separated selection from evaluation for this reason. Even so, only independent replication can establish the direction and specificity of the effect. Within the present data, the signal appears to be locus-specific. Unsupervised anomaly detection, which was driven by other genes, did not recover grandiosity from genotype outlier status, and the effect itself was confined to grandiosity. A marker of overall illness burden would have been associated with several symptom dimensions.

Grandiosity is common in psychosis and contributes to impaired insight, treatment non-adherence, risk-taking, and forensic risk (16), so a biological correlate would be of clinical use. The effect is small. Its interest lies in its pattern. The effect is recessive, it is confined to a single symptom dimension, and it does not track overall severity. It suggests that grandiosity has a partly constitutional, dimension-specific substrate that is separable from general illness severity. That in turn supports the move from diagnosis-level immune associations towards symptom-dimension biology, in line with transdiagnostic mappings of inflammatory genetics to specific symptoms (10). Mechanistically, it points to the interaction between anti-inflammatory cytokines and dopaminergic tone as a candidate substrate for symptoms of activation, separate from the link between inflammation and anhedonia that dominates the deficit syndrome (18).

### 4.1 Limitations

The result is based on a single cohort of one ancestry, with internal validation only. Independent replication in other populations and with other instruments, ideally pre-registered and at consortium scale, is the necessary next step. Related to this, population stratification cannot be formally excluded without genome-wide markers. Three features of the study reduce the scope for stratification: the cohort was recruited at a single site from one population, no marker or panel principal component separated cases from controls, and the association is within-case. None of them removes it.

The design is also cross-sectional and based on lifetime ratings. It therefore cannot relate the inherited set-point to the immune state of acute illness, which would require prospective cohorts with serial measurement of symptoms and cytokines. For the same reason, the study is mechanistically distant. Germline genotype indexes a tendency to produce a cytokine. We measured neither interleukin-10 protein nor its expression, and the functional effect of the variants was not tested. Direction and mechanism therefore remain inferred until functional work and fine-mapping, together with genome-wide data allowing formal ancestry adjustment, can establish them. The effect itself is small and depends on a recessive contrast in a minority carrier group, so the estimates are imprecise and require adequately powered replication; the split-half reproducibility of r = 0.36 likewise shows consistency of sign only.

The phenotype is a further constraint. The outcome is a compact checklist construct without severity or course, whereas grandiosity extends across mania and bipolar disorder and into the externalizing spectrum, where grandiose-manipulative traits form a recognized dimension (43). Richer, prospective, transdiagnostic phenotyping would sharpen the measure and test generalizability. Grandiosity was rated in 27% of patients and grandiose delusions in 22%, both below the approximately one half reported for psychosis samples (16), as would be expected for lifetime OPCRIT ratings in a chronic cohort. The binary contrast therefore rested on 133 events. A sample with more grandiosity would have more power, and whether the association holds in first-episode or affective psychosis is untested. Unmeasured clinical factors, including medication, chronicity, substance use, and metabolic state, influence both immune tone and symptoms and were not fully modeled. Treatment status at assessment in particular was not recorded. OPCRIT lifetime ratings record whether grandiosity was ever present and do not measure its intensity under treatment. The effect held in patients whose psychotic symptoms did and did not resolve on neuroleptics. Even so, an effect of medication on the ratings cannot be excluded. Underlying all of this is the poor replication record of candidate-gene findings in psychosis (44), which the present design reduces but does not remove, and which is why the result is framed as hypothesis-generating throughout.

## 5 Conclusion

Interleukin genotype as a whole did not predict grandiosity. Within the panel, the IL10 −819/−592 promoter locus was associated with grandiosity and with no other symptom dimension. If this finding is replicated, the immune contribution to psychosis may be better sought at the level of individual symptoms than at the level of diagnosis. An inherited anti-inflammatory set-point may then be one factor that determines which symptoms a patient develops.

## Data Availability

The datasets presented in this article are not readily available because they contain sensitive patient genotype and clinical data governed by the original participant consent and ethics approval. The analysis code is available at https://github.com/maksbrzezicki/il10-grandiosity. Requests to access the datasets should be directed to the corresponding author, MB (mbrzezicki@neurologicalsociety.org).
