# Supplementary material for: Interleukin pathway genotype and the grandiosity dimension of psychosis: a machine learning gene set analysis with unsupervised anomaly detection controls
Source: Front Immunol. 2026 Oct 16;17:1936750. doi: 10.3389/fimmu.2026.1936750 (PMC13719335; doi:10.3389/fimmu.2026.1936750)
Supplement: Supplementary file 1 [file DataSheet1.docx]

Supplementary Material

Maksymilian A. Brzezicki, Przemysław T. Zakowicz, Paweł Kapelski, Monika Wilkość-Dębczyńska, Aleksandra Rajewska-Rager, Maria Skibińska

Interleukin pathway genotype and the grandiosity dimension of psychosis: a machine learning gene set analysis with unsupervised anomaly detection controls

All tables are produced by the notebook revision_analyses.ipynb from the analysis sample of 489 patients (genotype-only controls, n = 521, where stated). Gene symbols denote genes and their markers; IL10 −819/−592 denotes the locus formed from rs1800871 and rs1800872. p, p value; q, Benjamini-Hochberg false-discovery-rate q value; MAF, minor-allele frequency; HWE, Hardy-Weinberg equilibrium; OR, odds ratio; CI, confidence interval; SKAT, sequence kernel association test; LD, linkage disequilibrium.

**Supplementary Table S1. Marker-level quality control and case-control allele frequencies for the 25 single-nucleotide polymorphisms.** Counted allele is the minor allele in the analysis sample, on which dosages (0, 1, 2) are based. Call rate is in the 490 linked cases. HWE p is from a chi-square goodness-of-fit test in cases and, separately, in the 521 controls. The allelic OR compares minor-allele frequency in cases with controls; q is the BH-adjusted p across the 25 SNPs. The IL1RN 86 bp variable-number tandem repeat (called in 484 of 490 cases) is encoded as the count of the short (two-repeat) allele and is not shown.

| gene | SNP | position | counted allele | call rate, cases | MAF cases | MAF controls | HWE p cases | HWE p controls | allelic OR (case vs control) | allelic p | allelic q (BH, 25 SNPs) |
| --- | --- | --- | --- | --- | --- | --- | --- | --- | --- | --- | --- |
| IL1A | rs1800587 | -889 | A | 484/490 (98.8%) | 0.284 | 0.294 | 0.665 | 0.249 | 0.95 | 0.6193 | 0.91 |
| IL1A | rs17561 |  | A | 481/490 (98.2%) | 0.282 | 0.292 | 0.852 | 0.175 | 0.95 | 0.6125 | 0.91 |
| IL1B | rs1143643 |  | T | 488/490 (99.6%) | 0.388 | 0.385 | 0.148 | 0.886 | 1.01 | 0.8734 | 0.91 |
| IL1B | rs16944 | -511 | A | 488/490 (99.6%) | 0.339 | 0.334 | 0.404 | 0.291 | 1.02 | 0.8187 | 0.91 |
| IL1B | rs1143623 |  | G | 488/490 (99.6%) | 0.291 | 0.28 | 0.881 | 0.227 | 1.06 | 0.5873 | 0.91 |
| IL1B | rs1143633 |  | T | 482/490 (98.4%) | 0.384 | 0.38 | 0.177 | 0.59 | 1.02 | 0.8652 | 0.91 |
| IL1B | rs1143627 | -31 | G | 487/490 (99.4%) | 0.345 | 0.338 | 0.428 | 0.46 | 1.03 | 0.7597 | 0.91 |
| IL1RN | rs419598 |  | C | 483/490 (98.6%) | 0.294 | 0.265 | 0.784 | 0.198 | 1.16 | 0.1484 | 0.569 |
| IL1RN | rs315952 |  | C | 488/490 (99.6%) | 0.375 | 0.355 | 0.484 | 0.165 | 1.09 | 0.354 | 0.804 |
| IL1RN | rs9005 |  | A | 484/490 (98.8%) | 0.296 | 0.292 | 0.104 | 0.586 | 1.02 | 0.8373 | 0.91 |
| IL1RN | rs4251961 |  | C | 482/490 (98.4%) | 0.314 | 0.352 | 0.896 | 0.36 | 0.85 | 0.0783 | 0.569 |
| IL6 | rs1800797 | -597 | A | 471/490 (96.1%) | 0.451 | 0.456 | 0.443 | 0.447 | 0.98 | 0.8371 | 0.91 |
| IL6 | rs1800795 | -174 | C | 490/490 (100.0%) | 0.461 | 0.459 | 0.966 | 0.577 | 1.01 | 0.9311 | 0.931 |
| IL6R | rs2228145 | Asp358Ala | C | 483/490 (98.6%) | 0.381 | 0.332 | 0.861 | 0.221 | 1.24 | 0.022 | 0.551 |
| IL6R | rs4537545 |  | T | 487/490 (99.4%) | 0.375 | 0.336 | 0.906 | 0.274 | 1.18 | 0.0689 | 0.569 |
| IL6R | rs4845617 |  | A | 486/490 (99.2%) | 0.361 | 0.355 | 0.787 | 0.813 | 1.03 | 0.7583 | 0.91 |
| IL10 | rs1800871 | -819 | A | 486/490 (99.2%) | 0.226 | 0.25 | 0.586 | 0.203 | 0.88 | 0.2049 | 0.569 |
| IL10 | rs1800872 | -592 | T | 458/490 (93.5%) | 0.225 | 0.25 | 0.447 | 0.193 | 0.87 | 0.2003 | 0.569 |
| IL10 | rs1800896 | -1082 | C | 488/490 (99.6%) | 0.47 | 0.446 | 0.707 | 0.724 | 1.1 | 0.2816 | 0.704 |
| IL10 | rs1800890 | -3575 | T | 486/490 (99.2%) | 0.4 | 0.372 | 0.976 | 0.447 | 1.13 | 0.1889 | 0.569 |
| IL10 | rs6676671 |  | A | 483/490 (98.6%) | 0.4 | 0.371 | 0.867 | 0.55 | 1.13 | 0.1876 | 0.569 |
| IL10RA | rs2229113 |  | A | 483/490 (98.6%) | 0.336 | 0.34 | 0.733 | 0.238 | 0.98 | 0.8596 | 0.91 |
| IL10RA | rs3135932 |  | G | 486/490 (99.2%) | 0.216 | 0.204 | 0.128 | 0.726 | 1.08 | 0.4981 | 0.91 |
| TGFB1 | rs1800469 | -509 | A | 483/490 (98.6%) | 0.336 | 0.353 | 0.248 | 0.506 | 0.93 | 0.4287 | 0.893 |
| TGFB1 | rs1800470 | +869 (Leu10Pro) | G | 482/490 (98.4%) | 0.392 | 0.425 | 0.082 | 0.693 | 0.87 | 0.1404 | 0.569 |

**Supplementary Table S2. Pairwise linkage disequilibrium (r²) between markers within each gene set.** r² is the squared Pearson correlation of minor-allele dosage vectors over pairwise-complete patients in the analysis sample (n). Pairs with r² above 0.8 are flagged; in the LD-pruned sensitivity analysis (Table S3) the second member of each flagged pair was dropped.

| gene | marker 1 | marker 2 | n | r2 | flag (r2 > 0.8) |
| --- | --- | --- | --- | --- | --- |
| IL1A | rs1800587 | rs17561 | 475 | 1 | yes |
| IL1B | rs1143643 | rs16944 | 486 | 0.186 |  |
| IL1B | rs1143643 | rs1143623 | 486 | 0.14 |  |
| IL1B | rs1143643 | rs1143633 | 480 | 0.977 | yes |
| IL1B | rs1143643 | rs1143627 | 485 | 0.183 |  |
| IL1B | rs16944 | rs1143623 | 486 | 0.771 |  |
| IL1B | rs16944 | rs1143633 | 480 | 0.18 |  |
| IL1B | rs16944 | rs1143627 | 485 | 0.981 | yes |
| IL1B | rs1143623 | rs1143633 | 480 | 0.142 |  |
| IL1B | rs1143623 | rs1143627 | 485 | 0.784 |  |
| IL1B | rs1143633 | rs1143627 | 479 | 0.177 |  |
| IL1RN | VNTR | rs419598 | 477 | 0.849 | yes |
| IL1RN | VNTR | rs315952 | 482 | 0.067 |  |
| IL1RN | VNTR | rs9005 | 478 | 0.502 |  |
| IL1RN | VNTR | rs4251961 | 476 | 0.186 |  |
| IL1RN | rs419598 | rs315952 | 481 | 0.071 |  |
| IL1RN | rs419598 | rs9005 | 479 | 0.559 |  |
| IL1RN | rs419598 | rs4251961 | 477 | 0.209 |  |
| IL1RN | rs315952 | rs9005 | 483 | 0.235 |  |
| IL1RN | rs315952 | rs4251961 | 481 | 0.16 |  |
| IL1RN | rs9005 | rs4251961 | 476 | 0.141 |  |
| IL6 | rs1800797 | rs1800795 | 471 | 0.963 | yes |
| IL6R | rs2228145 | rs4537545 | 481 | 0.915 | yes |
| IL6R | rs2228145 | rs4845617 | 479 | 0.024 |  |
| IL6R | rs4537545 | rs4845617 | 483 | 0.027 |  |
| IL10 | rs1800871 | rs1800872 | 455 | 0.988 | yes |
| IL10 | rs1800871 | rs1800896 | 485 | 0.253 |  |
| IL10 | rs1800871 | rs1800890 | 483 | 0.19 |  |
| IL10 | rs1800871 | rs6676671 | 479 | 0.19 |  |
| IL10 | rs1800872 | rs1800896 | 458 | 0.264 |  |
| IL10 | rs1800872 | rs1800890 | 456 | 0.195 |  |
| IL10 | rs1800872 | rs6676671 | 452 | 0.197 |  |
| IL10 | rs1800896 | rs1800890 | 484 | 0.745 |  |
| IL10 | rs1800896 | rs6676671 | 481 | 0.745 |  |
| IL10 | rs1800890 | rs6676671 | 480 | 0.996 | yes |
| IL10RA | rs2229113 | rs3135932 | 481 | 0.466 |  |
| TGFB1 | rs1800469 | rs1800470 | 477 | 0.764 |  |

**Supplementary Table S3. Gene-set screen under alternative SKAT kernels and weightings.** Each cell gives the per-gene SKAT p (exact Davies distribution) with the BH q across the eight genes in parentheses. Main analysis: linear kernel, equal weights, minor-allele dosages. Beta(1,25): each marker weighted by the Beta(1,25) density of its minor-allele frequency, rescaled to a maximum of 1. LD-pruned: linear kernel, equal weights, after dropping the second member of every within-gene pair with r² above 0.8 (Table S2). Standardised: each marker scaled to unit variance. All tests are adjusted for sex, age, and age at onset against the rank inverse-normal grandiosity score.

| gene | unweighted linear (main) | Beta(1,25) MAF-weighted | LD-pruned (r2 > 0.8), unweighted | standardised dosages |
| --- | --- | --- | --- | --- |
| IL1A | 0.6108 (q 0.814) | 0.6120 (q 0.721) | 0.5908 (q 0.847) | 0.6110 (q 0.815) |
| IL1B | 0.4948 (q 0.814) | 0.2874 (q 0.702) | 0.5042 (q 0.847) | 0.4910 (q 0.815) |
| IL1RN | 0.5444 (q 0.814) | 0.3509 (q 0.702) | 0.6256 (q 0.847) | 0.5394 (q 0.815) |
| IL6 | 0.8142 (q 0.814) | 0.8651 (q 0.865) | 0.9687 (q 0.969) | 0.8146 (q 0.815) |
| IL6R | 0.5953 (q 0.814) | 0.5857 (q 0.721) | 0.6355 (q 0.847) | 0.5963 (q 0.815) |
| IL10 | 0.0028 (q 0.023) | 0.0003 (q 0.002) | 0.0052 (q 0.042) | 0.0012 (q 0.010) |
| IL10RA | 0.4375 (q 0.814) | 0.2130 (q 0.702) | 0.4375 (q 0.847) | 0.3910 (q 0.815) |
| TGFB1 | 0.8109 (q 0.814) | 0.6305 (q 0.721) | 0.8109 (q 0.927) | 0.8080 (q 0.815) |

**Supplementary Table S4. Distribution of the composite grandiosity score (OPCRIT item 56 plus item 57) in the analysis sample (n = 489).** Item 57 (grandiose delusions) was rated above zero in 110 patients (22.5%) and item 56 (inflated self-esteem) in 100 (20.4%).

| grandiosity score | n | % |
| --- | --- | --- |
| 0 | 356 | 72.8 |
| 1 | 18 | 3.7 |
| 2 | 56 | 11.5 |
| 3 | 8 | 1.6 |
| 4 | 51 | 10.4 |

**Supplementary Table S5. IL10 −819/−592 copy number by grandiosity score.** Counts of patients at each score for 0, 1, and 2 copies of the shared −819/−592 minor-allele haplotype (n = 454 with the locus called). These aggregate counts are sufficient to recompute the recessive estimates in Section 3.3.

| IL10 -819/-592 copies | 0 | 1 | 2 | 3 | 4 | any grandiosity, n (%) | mean score | n |
| --- | --- | --- | --- | --- | --- | --- | --- | --- |
| 0 | 209 | 10 | 27 | 6 | 22 | 65 (24) | 0.62 | 274 |
| 1 | 108 | 5 | 19 | 0 | 22 | 46 (30) | 0.85 | 154 |
| 2 | 9 | 2 | 8 | 1 | 6 | 17 (65) | 1.73 | 26 |

**Supplementary Table S6. Autoencoder sensitivity to bottleneck width and weight penalty.** Architecture 26-16-k-16-26 with k = 2, 4, or 8, rectified-linear activations, Adam optimiser, L2 penalty alpha, up to 3000 iterations, fixed seed. In-sample mean squared reconstruction error on standardised dosages; out-of-sample error under five-fold cross-validation; PCA error with the same number of components; and the Spearman correlation of the autoencoder anomaly score with grandiosity. The main analysis uses k = 4 and alpha = 0.001; the ensemble without the autoencoder gave rho = -0.023 (p = 0.62).

| bottleneck | alpha | in-sample MSE | out-of-sample MSE | PCA MSE (same k) | rho AE score vs grandiosity |
| --- | --- | --- | --- | --- | --- |
| 2 | 0.001 | 0.797 | 0.834 | 0.684 | -0.007 |
| 2 | 0.01 | 0.8 | 0.835 | 0.684 | -0.005 |
| 4 | 0.001 | 0.478 | 0.536 | 0.502 | -0.06 |
| 4 | 0.01 | 0.476 | 0.536 | 0.502 | -0.06 |
| 8 | 0.001 | 0.23 | 0.283 | 0.223 | -0.032 |
| 8 | 0.01 | 0.221 | 0.285 | 0.223 | -0.032 |
